# Extensive pericardial calcification secondary to radiotherapy, causing mixed constrictive-restrictive pathology

**DOI:** 10.1259/bjrcr.20170036

**Published:** 2017-07-29

**Authors:** Miguel Paniagua González, María Luisa Sánchez Alegre

**Affiliations:** ^1^Radiology Department, Hospital General Universitario Gregorio Marañón, Madrid, Spain; ^2^Radiology Department, Hospital General Universitario Gregorio Marañón, Madrid, Spain

## Abstract

This report presents the case of a patient who suffered from a mediastinal neuroblastoma in his childhood (in 1977), having been treated by surgery, chemotherapy and radiotherapy. As a result, he developed multiple calcifications in the atria walls, interatrial septum, right ventricular free wall, mitral and aortic valves and pericardium, triggering a mixed constrictive and restrictive pathology.

## Case report

We report the case of a 41-year-old male patient who suffered from a mediastinal neuroblastoma in his childhood (in 1977), having been treated by surgery, chemotherapy and radiotherapy.

At first, he was admitted to his referral hospital, presenting an episode of progressive dyspnea and angina pectoris (NYHA class III). A thoracic X-ray was performed, showing an extensive pericardial calcification without remarkable anomalies in the lung parenchyma.

**Figure 1.**
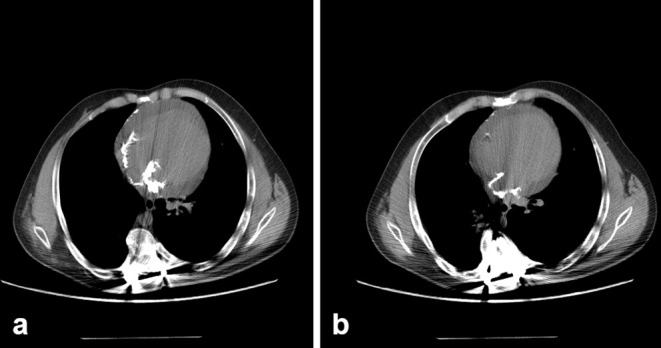
Non-enhanced CT, showing extensive pericardial and atrial calcifications.

A thoracic CT revealed the presence of multiple calcifications in the atria walls, interatrial septum, right ventricular free wall, mitral and aortic valves and pericardium. A significant pericardial effusion was also present (Figure 1).

The patient got worse, displaying signs of cardiac tamponade. Thus a pericardiocentesis was necessary. Nonetheless, the symptoms persisted even after reducing the pericardial effusion. Considering a pericardial window surgery, the patient was referred to our hospital.

Upon his arrival, an electrocardiogram was performed, together with an echocardiography. It displayed the aforementioned calcifications, as well as a moderate–severe effusion (15–25 mm, surrounding the right ventricle free wall and its lower face), and protodiastolic movement of the interventricular septum.

Considering a mixed constrictive–restrictive pathology, an MRI was performed, acquiring axial DP and *T*_2_-STIR “black-blood” sequences, standard BALANCED Fast Field Echo sequences (axial, two-chamber view, four-chamber view and short-axis view), as well as delayed-enhancement and phase-contrast sequences (in aortic and pulmonary levels).

**Figure 2.**
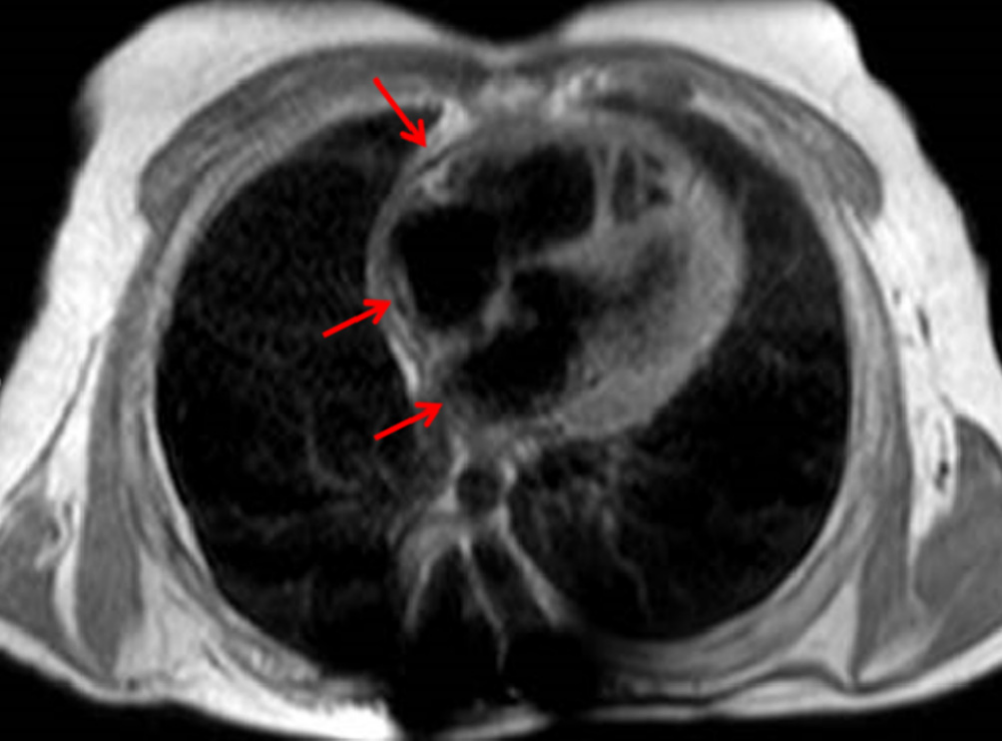
MRI axial “black-blood” sequence. Hypointense linear images (arrows) correspond to the calcifications shown in CT.

The images showed a segmental thickening of the pericardium and hypointense linear images in the interatrial septum and in the posterior wall of the atria (Figure 2), corresponding to the calcifications that had already been described in the prior CT.

The ventricles were not enlarged, and ejection fraction was normal (54%). Yet, there was a protodiastolic movement of the interventricular septum, evidencing constriction (Supplementary Video). The left atria was small sized (7 cm^2^), and the right atria was normal (19 cm^2^).

**Figure 3.**
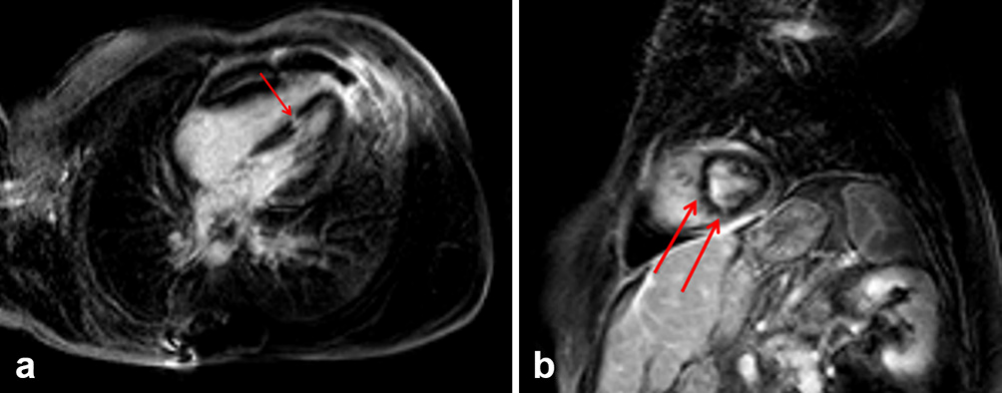
(a) MRI delayed-enhancement sequence (four-chamber view), showing patched enhancing area in the interventricular septum (arrow). (b) MRI delayed-enhancement sequence (short-axis view), showing patched enhancing area in the lower and anterior ventricle walls (arrows).

After the administration of paramagnetic contrast, the pericardium and the soft tissue of the anterior thoracic wall enhanced, probably as a result of the recent surgical manipulation. The delayed-enhancement sequence showed small patched enhancing areas, almost transmural, in the interventricular septum (Figure 3a) and in the lower and anterior ventricle walls (Figure 3b).

The MRI revealed a normal ventricular function, although with data of a mixed restrictive and constrictive physiology.

A few days after the surgery, the patient improved his symptoms, showed a correct heart rate and haemodynamic stability, so he was finally discharged.

## Discussion

Radiotherapy is a treatment for oncologic patients with increasingly satisfactory results, despite the well-known collateral damage caused to the healthy organs included in the field of radiotherapy. The radiotherapy equipment used decades ago did not allow the administration of the radiation on the affected anatomic area with today’s accuracy. The subsequent complications after the administration of radiotherapy depend on the irradiated area, the radiation dose and the fractionation of the dose. The latency of radiotherapy-associated cardiac effects ranges from months for subclinical disorders such as pericarditis, to decades for clinical diseases such as coronary artery disease resulting in myocardial infarction. Still most patients remain asymptomatic.^1^

Cardiac diseases associated with radiation therapy include acute and delayed pericarditis, pericardial and myocardial fibrosis, coronary artery disease and valvular disease.

It is important to differentiate constrictive pericarditis from restrictive myocardiopathy. Although there is a predominance of right heart failure symptoms in both diseases, the treatment is quite different.

Pericarditis presents as a thickening of the pericardium (over 4 mm is considered pathological), with or without pericardial effusion,^2^ although in some cases, it may not be thickened. Acute pericarditis often resolves spontaneously, but if it becomes chronic it may lead to a constrictive pericarditis. The loss of the normal elasticity of the pericardial sac leads to impairment of ventricular filling in mid- and late-diastole. Even though this pathology has traditionally been related to tuberculosis, nowadays there are a number of diseases that may cause constrictive pericarditis as well, like viral infections, postoperative fibrosis or thoracic radiotherapy.

Constrictive pericarditis causes a significant thickening and fibrosis of the visceral and parietal pericardium, often showing adhesions to the adjacent myocardium. Pericardial calcifications may appear in 50% of the cases.^3^ These alterations lead to a low compliance of the pericardium, triggering diastolic dysfunction and right heart failure.

In addition to the thickening of the pericardium, there may be compression and flattening of the right ventricle free wall, atrial dilatation and an increase of the calibre of both the cava and suprahepatic veins. Flattening of the right ventricle free wall is also typical.^4^

For a proper diagnosis of constrictive pericarditis, the visualization of morphological changes in the pericardium is required, as well as the demonstration of the consequent physiological alterations.

In cine sequences, *protodiastolic movement of the interventricular septum *is very frequent. This is a very typical sign, consisting of a paradoxical movement of the septum towards the left ventricle during the diastole, due to the low compliance of ventricular walls (that can be caused by pericardial thickening or by a pericardial effusion—cardiac tamponade). Such movement is more evident during the inspiration, as the intrathoracic pressure reduces, leading to a major venous return while the right ventricle pressure increases. This finding may be very helpful to differentiate constrictive pericarditis from restrictive myocardiopathy (sensitivity 81%, specificity 100% and positive predictive value 100%).^5,6^

The diastolic dysfunction can be also be determined by registering the intraventricular volume during the diastole. With this method, we can obtain graphics of the quick filling of the ventricles in early diastole, followed by a plateau (when the chambers cannot distend anymore), showing the typical “dip-plateau” curves. This finding can also appear in advanced restrictive cardiomyopathy.

The treatment for constrictive pericarditis is pericardiectomy, and long-term survival is related to the underlying cause. In particular, postoperative survival in post-irradiation constrictive pericarditis is poor.^7^ It is also worth mentioning that a number of patients with constrictive pericarditis may not benefit from pericardiectomy because of associated myocardial compliance abnormalities (ie. restrictive cardiomyopathy), myocardial atrophy after prolonged constriction, or other myocardial processes. This emphasizes the need for a complete evaluation of the heart and pericardium.^8^

Restrictive cardiomyopathy is characterized by a non-dilated rigid ventricle, resulting in severe diastolic dysfunction and restrictive filling that produces haemodynamic changes similar to those in constrictive pericarditis In contrast, this case displays a pericardium that is not thickened. In fact, it is morphologically normal.

It involves a diverse number of diseases that lead to myocardium infiltration (amyloidosis, sarcoidosis, haemochromatosis, hypereosinophilia, tumour dissemination etc.).

Another important difference is that in constrictive pericarditis the four cardiac chambers are equally affected (as they are all surrounded by pericardium), whereas in restrictive cardiomyopathy there is a characteristic atrial dilatation, even if ventricles keep a normal size. This is due to the fact that the ventricles have a higher amount of muscular tissue than the atria. Therefore, they are more heavily impacted by myocardial infiltration. As a result, the ventricles show a lower compliance, the atria receive too much blood and finally their size increases significantly.^9^ On the other hand, most patients suffering myocardial damages after radiotherapy show interstitial fibrosis and microvascular lesions worsening the diastolic dysfunction.^10^

In most cases, a myocardial biopsy is required to determine the cause of restriction. MRI can help in the characterization, as some of these diseases display a different behaviour in the different MRI sequences, or different enhancement patterns after gadolinium administration.

Patients who suffer constrictive pericarditis may not present parietal enhancement.

Coronary angiography is frequently used to establish the distinction between constrictive and restrictive disease. In constrictive pericarditis, coronary angiography typically shows nearly equal levels of diastolic pressure in all cardiac chambers, as it is caused by a symmetrical pathological process around the entire heart. In restrictive cardiomyopathy, diastolic pressure also tends to equalize between left and right chambers, but it remains higher in the left ones. A discrepancy of more than 5 mm Hg may make more suitable the possibility of a restrictive process.

In our patient coronary angiography showed the following diastolic pressures: right atria (15 mm Hg), right ventricle (18 mm Hg), left atria (22 mm Hg) and left ventricle (24 mm Hg). Therefore, pressure levels were similar but with a difference of more than 5 mm Hg between left and right chambers, according to restrictive cardiomyopathy.

Probably it did not revealed signs of constriction because it was carried out several days after pericardial window surgery.

The treatment for restrictive cardiomyopathy is different according to the infiltrating disease, but they usually require pharmacological management. In eligible patients with intractable heart failure, cardiac transplantation should be performed. Restrictive cardiomyopathy due to radiation/chemotherapy has lower survival rates than other restrictive cardiomyopathy patients.

The patient we are reporting is quite peculiar for two reasons. Firstly, nowadays it is not easy to find a case of such extensive pericardial and myocardial calcifications, since they were caused by radiotherapy administered during a long period of time. Secondly, the extension of the calcifications was such that they affected the cardiac walls and the pericardium at the same time, thus leading to mixed constrictive and restrictive cardiopathy (an uncommon situation, since these two pathologies usually have different causes).

## Learning points

1. Alteration in radiotherapy field or targeted radiation, with avoidance and/or shielding of the heart, remains one of the most important tenets (or points) in the prevention of radiation- induced cardiac damage.
2. Constrictive pericarditis and restrictive cardiomyopathy lead to diastolic heart failure with normal (or almost normal) systolic function, and abnormal ventricular filling, resulting in similar clinical and haemodynamic features. Given their different therapeutic management, it is essential to draw a distinction between them. Although history and diagnostic tests can help differentiate the two entities, biopsy or surgical exploration may be occasionally required.

## Consent

Written informed consent for the case to be published (including images, case history and data) was obtained from the patient(s) for publication of this case report, including accompanying images.
